# Population-associated differences between the phase variable LPS biosynthetic genes of *Helicobacter pylori*

**DOI:** 10.1186/1471-2180-6-79

**Published:** 2006-09-18

**Authors:** Laurence Salaün, Nigel J Saunders

**Affiliations:** 1 Bacterial Pathogenesis and Functional Genomics Group, The Sir William Dunn School of Pathology, University of Oxford, South Parks Road, Oxford OX1 3RE, UK; 2 Laboratoire des Spirochètes, 28 rue du Docteur Roux, Institut Pasteur, 75724 Paris Cedex 15, France

## Abstract

**Background:**

Population structures are normally determined using genes under minimal functional selection. In this study we have assessed genes that are not always essential, show differences in alleles between strains, and are involved in the directly host-selectable phenotype of LPS biosynthesis.

**Results:**

Eight complete LPS biosynthesis genes, seven of which are associated with phase variation in some or all strains of *Helicobacter pylori*, have been sequenced and their divergence analyzed. The differences observed indicate that recombination within these genes largely reflects exchange between strains within the population lineages previously determined on the basis of MLST using housekeeping genes. This indicates that the differences that are used for MLST are likely to broadly associate with genes under functional selection, and differences in strain behaviour. However, instances of exchange between the subpopulations were identified, including the hpAfrica2 subpopulation. Further, there were other differences in gene complements and the chromosomal location of genes indicative of greater diversity within the population than is revealed by the available genome sequences and comparative genome hybridization studies.

**Conclusion:**

These results indicate that the described population structure based upon MLST is broadly a good basis for studying the biology of *H. pylori*, but that individual alleles may not follow these associations. As a consequence, when working in unsequenced strains, it is necessary to carefully check the presence, sequence, and distribution of any individual gene of interest.

## Background

The LPS of *Helicobacter pylori *is a primary host-interacting structure and several lines of evidence indicate that it is involved in host adaptation and virulence. Strains lacking the O-antigen have a reduced capacity to colonize the murine stomach [1], and various alternative extensions form structural motifs which are identical to human blood group antigens Lewis X, Y, and b [2-6], which may be involved in immune evasion and the ability to establish long term colonization [7,8]. LPS structures are also the context in which surface proteins are presented and different LPS phenotypes are capable of affecting the presentation and function of these proteins, for example influencing flagellum-mediated motility and urease activity [9].

Almost half of the LPS biosynthetic genes of *H. pylori *are phase variable. Phase variability is a consistent marker for genes involved in niche adaptation and immune evasion [10,11]. The phase variable repertoire of this species has been recently re-evaluated in a population-based survey [12], and the variability of these genes has been studied in a model of prolonged gastric colonization [13]. These studies have shown that the LPS biosynthetic genes are amongst the most abundant phase variable genes, are amongst those that display the greatest inter-strain differences in their repeats, and are the most dynamically variable during colonization. The sequencing used to assess the phase variable status of the genes in these previous studies was focussed upon the regions containing the repeats. This revealed that there were additional polymorphisms within the sequences flanking the repeats between strains, suggesting frequent recombination as a significant additional source of previously uncharacterized inter-strain variability within these genes, which was the stimulus for the study describe here.

The selection of sequences used for bacterial population studies and typing is essentially pragmatic. The selected target must show sufficient diversity to provide discrimination between unrelated strains. It must also be sufficiently stable to indicate when two strains are related over a biologically relevant period of evolutionary time. The dominant methodology used for studies of bacterial population genetics is currently MLST, and this has been recently applied successfully to *H. pylori *[14,15] revealing clear correlations between the population structure of the bacteria and the human migratory populations with which they are associated. *H. pylori *is one of the most recombinogenic species to have been studied to date [16-18] in which relatively small sections of DNA are exchanged. The MLST studies focus upon genes associated with core metabolic processes which are believed to be under minimal selective pressure for change.

What is currently undefined is the degree to which these sequences, which have been selected as a basis for strain typing because of their relative stability, reflect the patterns of exchange and recombination of more diverse genes that are potentially subject to functional selection for change. It may be that the population divisions do not reflect functional barriers to the free exchange of other genes in the global population, and that sequences directly affecting strain behaviour move more freely through the population. Equally, it may be the case that these populations that have been relatively geographically isolated are broadly similar in other ways and that this would be reflected in their allelic-variant gene complements as a whole. While the latter possibility is generally assumed to be more likely, it is important to specifically address this issue, because when strains are selected for functional and mutagenesis-based experiments it is necessary to know to what extent the findings are likely to be applicable to the wider bacterial population, especially in a highly panmictic species such as *H. pylori*. In this study, we have focussed upon eight LPS biosynthetic genes in which we have previously seen polymorphisms, seven of which are phase variable, to determine their diversity and the extent to which they are mobile within and between the major population subdivisions, using strains representative of the previously defined population structure.

## Results and Discussion

### Variations in gene complement and gene location

Our previous study of *H. pylori *phase variable genes used primer pairs designed to amplify repeats mediating phase variation that were located within and immediately adjacent to the coding sequences. This revealed some differences in gene complement between the strains and the presence of polymorphisms indicative of recombination [12]. The current study sought to investigate the diversity within the whole of the coding regions of the selected genes, and primers were designed to locations in the flanking regions of these genes – normally within the coding sequences of the adjacent genes. This led to the unanticipated finding that some of these genes, whilst previously shown to be present, are located in different, yet to be determined, chromosomal locations. This is not a consequence of failed amplification due to polymorphisms in primer target sites, or local reorganizations, because in some instances the sites in which the genes are located in the sequenced strains can be amplified, sequenced, and shown to be 'empty'. The data on gene complements and locations is summarized in Table 1.

Gene jhp820, an *rfaJ *homologue, is not present in strain 26695, nor in B225, JP9, and 162, which is consistent with our previous findings. In the current study the gene was not amplified using the flanking primers from a further 8 strains, although we know from our previous study that these strains possess this gene [12]. In these 8 cases short PCR products were obtained from this chromosomal locus corresponding to an empty site, as was obtained from strain 26695. Sequences of two representative products show the expected flanking regions and the absence of jhp820 in this location. This indicates that although this gene is present in these strains, it is located at a different chromosomal position.

In two strains (L133 and C164) sequencing of the complete jhp820-equivalent genes from this genomic location, using primers located within the flanking genes, revealed intact homopolymeric tracts sufficiently long to mediate phase variation, when in our previous study [12] these were found to be stabilized with an internal thymidine. This indicates that there are actually at least two copies of this gene in these strains, and that those from which the internal region was previously sequenced is located in a different genomic location. In strain J99 this gene is located between one of the *vacA *genes (jhp0819) and *fecE *(jhp0821, an iron transport associated gene). The primer pair used previously extended into the *vacA *gene because the homopolymeric tract is located close to the 5' end of the coding sequence. The *vacA *gene is present in multiple copies, with one gene and four and three paralogues annotated in strains 26695 and J99, respectively. One possible scenario is that *vacA *is variably associated with different *rfaJ *homologues in two or more of its different locations. Interestingly, the absence of gene jhp0820 in this location, or its replacement with another allele, is a feature shared by the strains of the hpLadakh and hpEastAsia populations. Because the PCR and sequencing data for this gene were obtained from only 11 of the 23 strains selected for this study, jhp0820 was not included in the population structure analyses that follow.

HP0217, encoding for a transferase involved in LPS biosynthesis, was not amplified from the predicted location in two strains: NQ367 (hpEurope) and CC42C (hpAfrica1), although we know this gene to be present on the basis of amplification and sequencing of its repeat-containing region in our previous study [12]. Primers located in the flanking genes, which encode hypothetical proteins, generated short products which did not contain this gene, indicating that this gene is not located in this genomic location in these two strains, and is also located in a different, currently unknown, site.

HP0619 is a homologue of *lex2B*, which is phase variable. Adjacent to this gene in strain J99, but not in the published sequence of strain 26695, is a related gene jhp0562 which is not phase variable. The population structure revealed by the gene jhp0562 is broadly similar to the one described on the basis of the MLST analysis. At least 16 strains possess both genes, and strain 26695 is unique within the studied collection in having only HP0619. Three strains: strains L67 (hpLadakh), VZ21 (hpEurope), and GU5 (hpEastAsia), only contained an orthologue of jhp0562. However, the situation in some strains is more complex. In strain JP9 (hpEastAsia), two PCR products were obtained. Both products were sequenced, which revealed the presence of two *lex2B *homologues in one locus (HP0619 and jhp0562 homologues), reflecting the more common reported state, and an additional different one (a jhp0562 homologue) from the other product. The sequences of all three genes are clearly distinct on the basis of polymorphisms. Taken together this indicates that there are at least two loci containing these homologues, suggesting a potential for substantial diversity in the synthesis of different LPS structures.

### Comparison of the population structures revealed by MLST and phase variable LPS biosynthetic gene sequences

The LPS genes are much more diverse than the housekeeping genes (Table 2). Higher genetic distances and lower nucleotide identities were found for the LPS genes than for the housekeeping genes. Moreover, the higher rate of non-synonymous substitution (Ka) found in LPS genes show that the purifying selection exerted on housekeeping genes is much higher than on the LPS genes. The main difference observed between the MLST tree, built with the 7 housekeeping genes sequences (total of 3.4 kb of sequence), and the LPS tree, built on the 7 LPS genes (the 8 LPS genes excluding jhp0820 representing a total of 7.3 kb of sequence), is the depth of the branches. This higher genetic diversity in the LPS biosynthesis genes is consistent with these genes being under different selective pressures to the housekeeping genes used for MLST.

**Figure 1:**
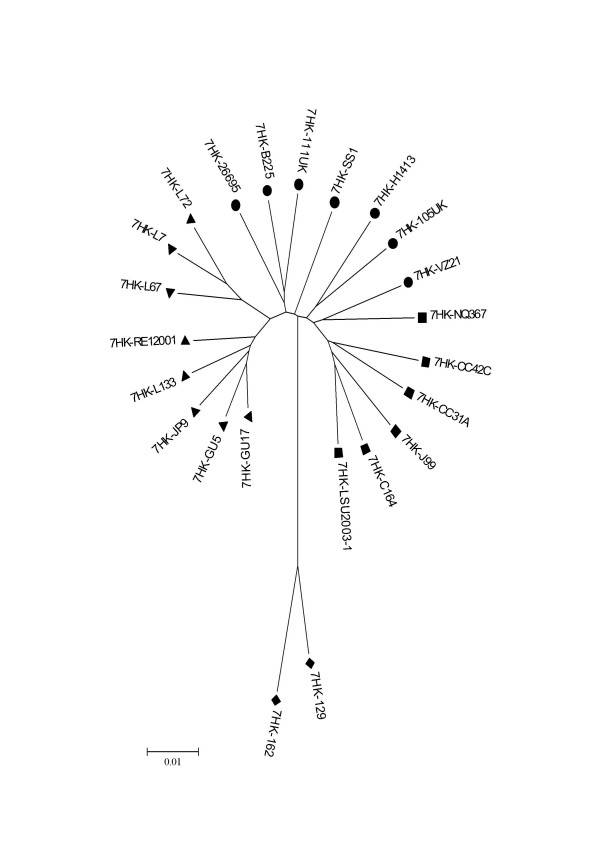
**Neighbour-joining tree based on MLST data**. All distances are based on Kimura two-parameter estimates and are to scale (scale bar, lower left). Population are coded such as: ▲ hpEastAsia; ▼ hpLadakh; ● hpEurope; ■ hpAfrica1 and ◆ hpAfrica2.

**Figure 2:**
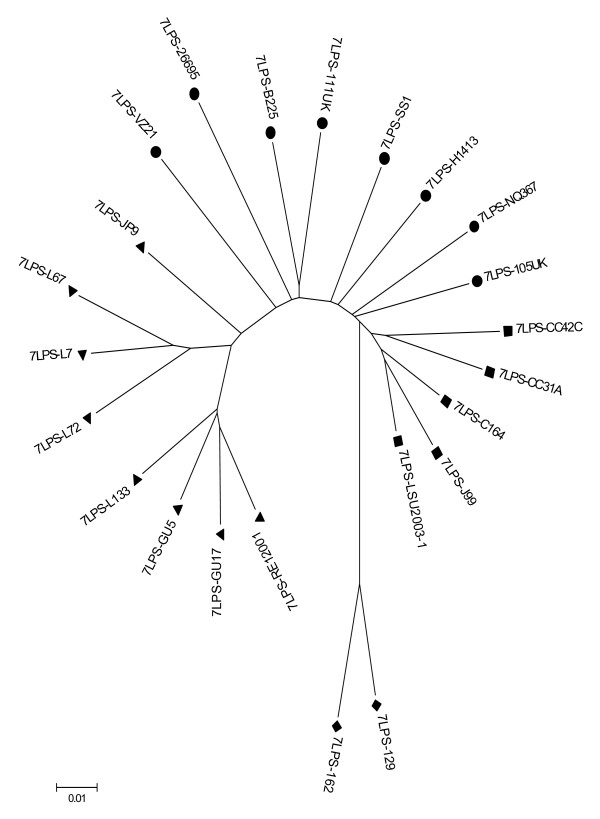
**Neighbour-joining tree based on LPS biosyntheticgenes sequencing data**. All distances are based on Kimura two-parameter estimates and are to scale (scale bar, lower left). Population are coded as in Figure 1.

The general pattern for all of the genes studied is that there are strong associations between the described MLST-based population subdivisions (Figure 1) and the differences in the seven phase variation-associated LPS biosynthetic genes (Figure 2). This pattern is clearest once the lengths of the phase variation-mediating repeats have been standardized and major indels removed, but is consistent for complete, repeat and indel standardized, and amino acid based analyses. It should be noted that the MLST tree presented here is different from the tree published previously [12] because the *vacA *sequence has been excluded from the current analysis. This is in part because we wished to compare the population structure based upon the core housekeeping genes under minimal selection, with that based upon the phase varied LPS biosynthetic genes. It is also because the *vacA *gene fragment used for MLST is strongly biased by the so-called s-region encoding the gene's leader peptide, and there is a strong correlation between the s-region (1 or 2) and the cagPAI status (positive or negative respectively) leading to a false phylogeny of the resulting supergene (Bodo Linz – personal communication).

There are clear areas of sequence divergence indicative of recombination, and the clustering shown within the trees of this study indicate that the predominant movement of sequences is within, rather than between, the population subdivisions. This indicates that the population structures, as defined using MLST, are likely to be broadly associated with relatively conserved similarities and differences between the strains with regard to their general characteristics, and that these subdivisions do, indeed, form a sound basis for the investigation of biological differences between different *H. pylori *strains.

The housekeeping gene-based MLST tree distinguishes 5 populations: hpEastAsia (boostrap value = 100), hpLadakh (bootstrap value = 90), hpAfrica1 (bootstrap value = 93), hpAfrica2 (bootstrap value = 100) which is rooted in hpEurope (Figure 1), which is consistent with the previous findings using these sequences. The tree based upon the combined LPS biosynthesis gene sequences group the strains from the previously defined sub-populations similarly (Figure 2), although the branch orders differ, and generally associates strains in the previously assigned sub-populations most closely. However, there are some differences between the two trees. Specifically, in the LPS biosynthesis gene-based tree, hpLadakh appears as a subpopulation of hpEastAsia as illustrated by the relative position of strain JP9, and hpAfrica2 is rooted between hpAfrica1 and hpEurope, rather than from the midst of the hpEurope population. From both trees, based upon the Maynard-Smith *et al*. model [19], *H. pylori *has a population consistent with a clonal epidemic structure, with clonal groupings (hpEastAsia, hpLadakh, hpAfrica1 and hpAfrica2) and a recombinant structure in hpEurope. When the sequence data of the 7 LPS genes and the 7 housekeeping genes were combined the resulting Neighbour-Joining tree had the same overall shape as the tree obtained with the MLST data combining the sequence of the 7 housekeeping genes with the *vacA *gene fragment.

With the exception of HP0379, all of the single gene-derived trees indicate that the hpAfrica2 population is the most divergent from the others, and that the hpEurope and hpAfrica1, and the hpEastAsia and hpLadakh populations are the most closely associated pairs of population subdivisions. This contrasts with the previously described attribution of the hpLadakh strains as part of the hpEurope population [14].

### The behaviour of individual genes in the population

The orthologues of HP0651, an alpha-1,3-fucosyltransferase, shows inter-strain differences in the presence of a long homopolymeric cytidine repeat, or a shorter hexamer which is not predicted to mediate phase variation. The presence of the longer and shorter repeats shows a clear division (bootstrap value = 100) within the population. This indicates that the emergence of longer repeats mediating phase variation is probably a relatively uncommon evolutionary event. It may also reflect the gene complements of a specific group of strains, in which phase variation of a particular gene may be less adaptively advantageous. The separation of the different alleles remains after the variable length repeats have been adjusted to a uniform length. The population grouping roughly follows the population grouping obtained with the MLST study, with hpAfrica1 strains constituting a cluster (bootstrap value = 80) and hpAfrica2 strains constituting a different cluster rooted in the hpEurope population.

The other alpha-1,3-fucosyltransferase sequenced, the orthologues of HP0379, shows the only example in this study of integration of alleles from hpAfrica2 with another part of the population. In this case the hpAfrica1 and hpAfrica2 alleles cluster together (bootstrap value = 100) indicating an exchange of this allele between these two populations, and that opportunities for exchange, either directly or indirectly, with this most-divergent of *H. pylori *populations do occur. HpLadakh strains constitute a cluster (bootstrap value = 100) distinct from the hpEastAsia population and the hpEurope population. This illustrates that even though the previous MLST-based population study robustly demonstrated that this population had a prolonged genetic isolation from the rest of the *H. pylori *population [14], these strains still have a degree of genetic connectedness with the wider *H. pylori *gene pool.

**Figure 3:**
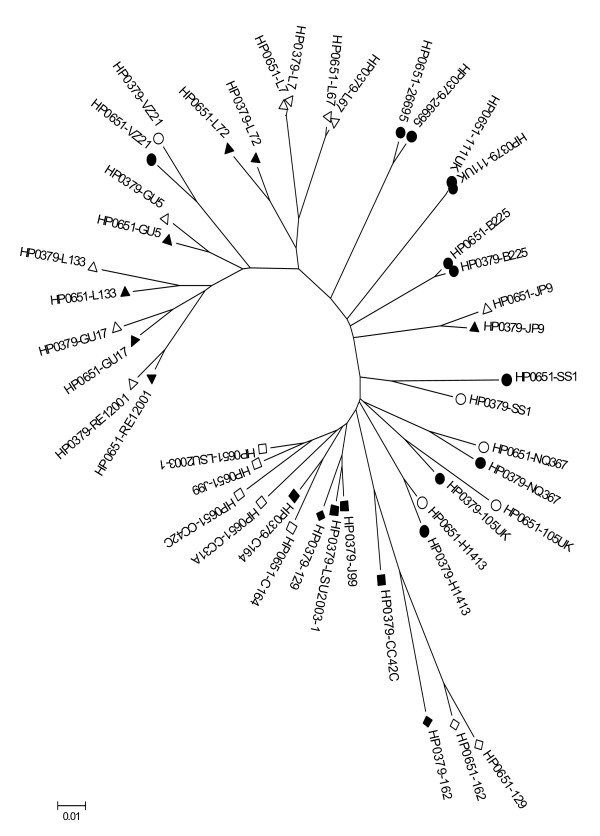
**Neighbour-joining tree based on combined sequencing data of the two alpha-1,3-fucosyltransferase genes (HP0651 and HP0379)**. All distances are based on Kimura two-parameter estimates and are to scale (scale bar, lower left). Population are coded as in Figure 1; Solid shapes indicate phase variable alleles and empty shapes indicate non-phase variable alleles.

When data for the genes HP0651 and HP0379 are combined (Figure 3), the comparatively recent transfer of HP0379 within the African populations is readily apparent. While in the wider non-African populations both genes have been present for sufficient time for their distribution to show a common strain-associated pattern of divergence, the strain associations break down in the African populations, where these genes are clustered primarily on the basis of the similarities between the genes rather than on their divergence since acquisition by individual strains. HpAfrica1 and hpAfrica2 strains are part of a single cluster (bootstrap value = 100), hpEastAsia strains constitute a cluster (bootstrap value = 97) distinct from hpLadakh (bootstrap value = 94), whereas hpEurope strains are placed all around the tree, without forming a true group. It is usually, but not always, the case that one of the two alpha-1,3-fucosyltransferase genes is phase variable, while the other is not. Among the 8 strains of the hpAfrica1 and hpAfrica2 populations, HP0379 is not phase variable and HP0651 is phase variable. In two strains of hpLadakh population (L7 and L67) neither of these genes is phase variable, and in four strains of hpEurope population (26695, B225, H1413, 111UK) both are phase variable. The functional consequences of which, or both, of these genes is phase varied has yet to be determined.

**Figure 4:**
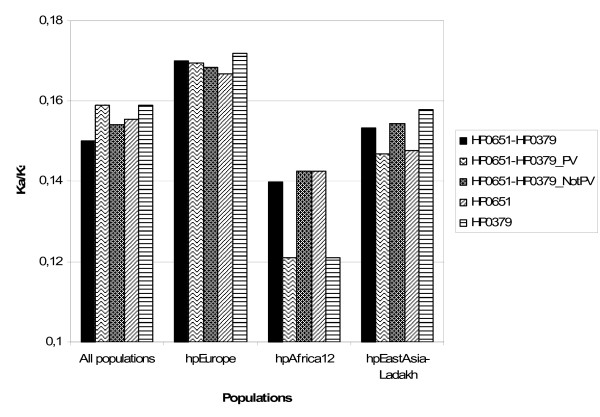
**Non-synonymous/synonymous substitution rate (Ka/Ks) analysis of the two alpha-1,3-fucosyltransferase genes (HP0651, HP0379)**. Considering the small number of strains in each population, hpAfrica1 and hpAfrica2 populations were grouped together in a "hpAfrica1-2" cluster, and hpEastAsia and hpLadakh populations were together in a "hpEastAsia-Ladakh" cluster.

The neighbour-joining trees for HP0651 and HP0379 show different patterns of evolution in the hpAfrica1 and hpAfrica2 populations compared to the patterns observed in the other populations. In order to better understand the differences, these populations were studied separately. Considering the relatively small number of strains used in this study from each population, hpEastAsia and hpLadakh were grouped together (referred to as "hpEastAsia-Ladakh"), which was suggested to be reasonable by trees built using the other LPS genes sequences. On the same basis, hpAfrica1 and hpAfrica2 populations were grouped together (referred to as "hpAfrica1-2"). The genetic distances in the "hpAfrica1-2" and "hpEastAsia-Ladakh" groups are shorter than those in the hpEurope population, suggesting that the strains in the so-called hpEurope population are more divergent than the strains from hpEastAsia and hpLadakh, or than the strains from hpAfrica1 and hpAfrica2. The genetic distances in hpEurope (d_HP0619-HP0379 _= 0.11 ± 0.03) are as high as the genetic distances in all of the populations considered together (d_HP0619-HP0379 _= 0.12 ± 0.03). This is consistent with a recombinant population structure for hpEurope, whereas the shorter genetic distances in both hpAfrica1-2 (d_HP0619-HP0379 _= 0.09 ± 0.04) and hpEastAsia-Ladakh (d_HP0619-HP0379 _= 0.09 ± 0.03) groupings are consistent with clonal populations. Nearly identical synonymous substitutions (Ks) frequencies were found for HP0651 and HP379 in hpEurope (Ks_HP0619 _= 0.46 ± 0.07; Ks_HP0379 _= 0.42 ± 0.16) and hpEastAsia-Ladakh (Ks_HP0619 _= 0.34 ± 0.07; Ks_HP0379 _= 0.35 ± 0.09), suggesting that the interval since their divergence from an ancestor is similar. In contrast, the different Ks frequencies found in hpAfrica1-2 (Ks_HP0619 _= 0.37 ± 0.20; Ks_HP0379 _= 0.29 ± 0.17) suggest that HP0651 and HP0379 have a different pattern of evolution. Non synonymous/synonymous substitution rate analysis of HP0651 and HP0379 suggests that hpAfrica1-2 has undergone a higher purifying selection than hpEurope and hpEastAsia-Ladakh (Figure 4). Ka/Ks analysis also shows that, in the hpAfrica1-2 population, the selective pressure is greater on HP0379, the phase variable alpha-1,3-fucosyltransferase gene, than on HP0651, the non-phase variable allele, whereas in hpEurope and hpEastAsia-Ladakh the selective pressure exerted on HP0651 and HP0379 is similar.

HP0208 shows a different pattern of subpopulation exchange, in that it has a pattern which indicates recombination of this gene between the hpAfrica1 and hpEurope lineages. This gene is absent from the hpLadakh strains, but otherwise has a pattern reflecting the general population subdivisions, with hpEastAsia (bootstrap value = 98) and hpAfrica2 constituting two clusters whereas hpAfrica1 and hpEurope strains constitute a background population. Similarly, HP0217 and HP0093 show a generally typical pattern, but with some evidence of exchange from the hpEastAsia and hpLadakh populations to some hpEurope strains. HP0619 and jhp0562 when considered independently show a generally typical pattern as well, but when these two genes are considered together, they show evidence of genetic exchange between hpLadakh and hpEastAsia.

Considering the overall patterns of all of the genes (Figure 2), and the different patterns illustrated by HP0379, HP0208, HP0217, and HP0093, there is a general picture in which these populations, hpEurope, hpEastAsia, hpLadadh, hpAfrica1 and hpAfrica2, are genuinely ecologically separate in many regards and as reflected by their general allelic compositions, but there are occasional bridging points which have allowed for the exchange of genes between these subpopulations. The nature of these exchanges, and that they differ on a gene-by-gene basis is of relevance to those designing and interpreting functional studies, and highlights the need to specifically address these issues of relatedness at both a population and gene-specific level when selecting representative strains for such projects.

## Conclusion

The MLST-based population subdivisions appear to broadly represent the allelic gene complements of the population in other genes outside of those associated with core 'housekeeping' metabolic functions. Therefore the described population subdivisions are a good basis for studying the biology of *H. pylori*, although the relatedness of the population subdivisions may be slightly different from those currently reported. The depth of neighbour joining tree branches are longer for the LPS biosynthesis genes than for the housekeeping genes reflecting their faster evolution. The stable association of long and short repeats within the population suggests that emergence of phase variable repeats is probably a relatively uncommon evolutionary event. Exchange of genes between hpAfrica2 and the more closely related populations can occur, or these populations can acquire genes from or through a common source. However, there are indications that there is considerable additional complexity in the repertoire and number of the LPS biosynthetic genes, and their genetic locations, which can vary significantly between individual strains. Notably, this type of diversity is not reflected in MLST or comparative genome hybridization studies that have been reported to-date. This diversity is poorly reflected by the limited number of currently available genome sequences, and it would be wise to check the presence, sequence, and distribution of any gene of interest when working in non-sequenced strains.

## Methods

### Bacterial strains and growth conditions

Twenty-three *H. pylori *strains were selected representing diverse ethnic groups and countries of origin [14], including the sequenced strains 26695 [20] and J99 [21], and the mouse-adapted strain SS1 [22] (Table 1). Culture conditions were as described previously [12].

### Amplification and sequencing of the phase variable LPS genes

DNA was prepared from plate cultures using the AquaPure Genomic DNA Isolation Kit (BioRad) according to the manufacturer's instructions. Seven phase variable LPS biosynthetic genes, encoding for two alpha-1,3-fucosyltransferases (HP0651 and HP0379), an alpha-1,2-fucosyltransferase (HP0093), a glycosyltransferase homologue of Lex2B (HP0619), two alpha-1,2-glycosyltransferase homologues of RfaJ (HP0208 and jhp0820), and a beta-1,4-N-acetylgalactoamyl transferase (HP0217), and a non-phase variable LPS biosynthetic gene (jhp0652, related to HP0619) were amplified and sequenced. Primers were designed using the published sequences of *H. pylori *strains 26695 [20] and J99 [21] [See Additional file 1]. PCRs were carried out using *Taq *DNA polymerase (Invitrogen) according to the manufacturer's instructions. PCR products were cleaned up and sequenced directly on both strands using the primers used for PCR. Automated sequencing used ABI Prism BigDye™ Terminator cycle sequencing, version 3.0 (Applied Biosystems) and was resolved on an ABI Prism 3100 DNA sequencer (Applied Biosystems).

### Phylogenetic and sequence analysis

Sequences were assembled using the program Gap4 from the Staden package [23]. Multiple alignments were performed using ClustalW [24], and then, manually edited using Seqlab from the Wisconsin Package, version 10.2 (Genetics Computer Group, Madison, Wisconsin) through the Oxford University Bioinformatics Centre. Analyses of nucleotide identity, genetic distances, and synonymous/nonsynonymous substitutions [25] were performed on multiple alignments using Swaap 1.0.0 (Pride, D.T. (2001). Swaap 1.0.0: a tool for analysing substitutions and similarity in multiple alignments. Distributed by the author, available at ) [26]. Neighbour-joining trees [27] based on distances with Kimura two-parameter estimates [28] were constructed, and their robustness was assessed using a bootstrapping procedure (500 repetitions) [29] using MEGA version 2.1 [30].

## List of abbreviations

LPS = lipopolysaccharides; MLST = multi-locus sequence typing; HK gene = housekeeping gene.

## Authors' contributions

LS carried out bacterial cultures, PCR and sequencing, sequence assembly and sequence analyses, contributed to experimental design of sequence analysis, and co-drafted the manuscript. NJS supervised the project and its experimental design, and co-drafted the manuscript. Both authors read and approved the final manuscript.

## Supplementary Material

Additional file 1 Primers used for amplification and sequencing of the LPS biosynthesis genes under study. This file contains the sequences of all the primers used in the study Click here for file

## Figures and Tables

**Table 1:** Presence and absence of the 8 LPS genes studied among 23 *H. pylori *strains

Strain name	Population	**HP0651**	**HP0379**	**HP0093**	**HP0619**	jhp0562*	**HP0208**	**jhp0820**	**HP0217**
**JP9**	hpEastAsia	○	●	●	●	+	●	absent	●
**GU17**	hpEastAsia	●	○	●	*missing*	*missing*	●	EMPTY	●
**GU5**	hpEastAsia	●	○	●	absent	+	●	EMPTY	●
**RE12001**	hpEastAsia	●	○	●	●	+	●	EMPTY	●
**L133**	hpEastAsia	●	○	●	●	+	●	⊙	●

**L7**	hpLadakh	○	○	●	*missing*	*missing*	absent	EMPTY	●
**L72**	hpLadakh	●	○	●	●	+	absent	EMPTY	●
**L67**	hpLadakh	○	○	●	absent	+	absent	EMPTY	●

**B225**	hpEurope	●	●	●	●	+	●	absent	●
**26695**	hpEurope	●	●	●	●	absent	●	absent	●
**SS1**	hpEurope	○	●	●	●	+	●	●	●
**H1413**	hpEurope	●	●	●	●	+	●	EMPTY	○
**NQ367**	hpEurope	○	●	●	●	+	●	●	EMPTY
**111UK**	hpEurope	●	●	●	●	+	●	EMPTY	●
**VZ21**	hpEurope	●	○	●	absent	+	●	●	●
**105UK**	hpEurope	○	●	●	●	+	●	●	●

**LSU2003-1**	hpAfrica1	○	●	●	●	+	●	●	●
**J99**	hpAfrica1	○	●	●	●	+	●	●	●
**C164**	hpAfrica1	○	●	●	●	+	●	⊙	●
**CC31C**	hpAfrica1	○	*missing*	●	●	+	●	●	●
**CC42C**	hpAfrica1	○	●	●	*missing*	*missing*	●	○	EMPTY

**129**	hpAfrica2	○	●	●	●	+	●	●	●
**162**	hpAfrica2	○	●	●	●	+	●	absent	●

Status of the genes is coded as followed:

●, gene present and harbouring the repeat

○, phase variable gene present but repeat absent or stabilized (i.e. repeat interrupted by one or more bases or a homopolymeric tract < 7 bp or dinucleotide tracts with < 5 repeats)

⊙, phase variable gene harbouring a repeat, whereas a stabilized repeat was found previously [12]

EMPTY, empty site, i.e. the phase variable gene was found using primers designed within the coding sequence in regions immediately adjacent to the phase variable tract [12], but the site is found to be empty in this study

*missing*, missing data, i.e. the PCR product was obtained but could not be sequenced absent, gene absent

* jhp0562 is a non phase variable homologue of HP0619 (+: jhp0562 present)

**Table 2:** Diversity within *H. pylori *LPS genes and housekeeping genes under study

gene	number of sequences compared	length compared (nt)*	nucleotide identity (%)	genetic distances^†^	Ks^‡^	Ka^‡^	Ka/Ks^‡^
**LPS genes**							
LPS supergene	23 × 7	7335	75.62 ± 14.62	0.07 ± 0.02	0.27 ± 0.08	0.05 ± 0.01	0.18 ± 0.02
HP0651	23	1065	88.49 ± 3.15	0.12 ± 0.03	0.49 ± 0.16	0.07 ± 0.02	0.15 ± 0.03
HP0379	22	1065	89.15 ± 3.22	0.11 ± 0.03	0.44 ± 0.15	0.07 ± 0.02	0.15 ± 0.03
HP0093	23	873	94.64 ± 1.93	0.05 ± 0.02	0.20 ± 0.08	0.02 ± 0.01	0.12 ± 0.03
HP0619	17	1119	91.29 ± 2.68	0.09 ± 0.03	0.29 ± 0.10	0.05 ± 0.02	0.20 ± 0.06
jhp0562	19 + 1^§^	999	92.20 ± 3.45	0.08 ± 0.03	0.26 ± 0.11	0.05 ± 0.03	0.19 ± 0.07
HP0208	20	1128	92.40 ± 2.27	0.08 ± 0.02	0.24 ± 0.08	0.05 ± 0.01	0.21 ± 0.04
jhp0820	11	1110	90.83 ± 8.33	0.10 ± 0.10	0.27 ± 0.24	0.10 ± 0.13	0.37 ± 0.15
HP0217	21	1086	94.40 ± 2.13	0.05 ± 0.02	0.19 ± 0.08	0.03 ± 0.01	0.18 ± 0.06
**HK genes**							
HK supergene	23	3402	95.31 ± 1.47	0.05 ± 0.02	0.21 ± 0.07	0.01 ± 0.01	0.06 ± 0.01

LPS supergene combines sequencing data of 7 LPS genes (i.e. the 8LPS genes under study except for jhp0820)

HK supergene combines sequencing data of the 7 housekeeping genes studied by MLST

* Lengths of the phase variation mediating repeats have been standardized and major indels removed

^† ^Molecular distances were based on Kimura two-parameter estimates [28]

^‡ ^Synonymous substitutions (Ks) and non-synonymous substitutions (Ka) were determined using the methods of Nei and Gojobori [25]

^§ ^in strain JP9, two PCR products were obtained, showing that jhp0562 was present in two different loci

## References

[B1] Logan SM, Conlan JW, Monteiro MA, Wakarchuk WW, Altman E (2000). Functional genomics of *Helicobacter pylori*: identification of a beta-1,4 galactosyltransferase and generation of mutants with altered lipopolysaccharide. Mol Microbiol.

[B2] Aspinall GO, Monteiro MA (1996). Lipopolysaccharides of *Helicobacter pylori *strains P466 and MO19: structures of the O antigen and core oligosaccharide regions. Biochemistry.

[B3] Aspinall GO, Monteiro MA, Pang H, Walsh EJ, Moran AP (1996). Lipopolysaccharide of the *Helicobacter pylori *type strain NCTC 11637 (ATCC 43504): structure of the O antigen chain and core oligosaccharide regions. Biochemistry.

[B4] Monteiro MA, Zheng PY, Appelmelk BJ, Perry MB (1997). The lipopolysaccharide of *Helicobacter mustelae *type strain ATCC 43772 expresses the monofucosyl A type 1 histo-blood group epitope. FEMS Microbiol Lett.

[B5] Monteiro MA, Chan KH, Rasko DA, Taylor DE, Zheng PY, Appelmelk BJ, Wirth HP, Yang M, Blaser MJ, Hynes SO, Moran AP, Perry MB (1998). Simultaneous expression of type 1 and type 2 Lewis blood group antigens by *Helicobacter pylori *lipopolysaccharides. Molecular mimicry between *H. pylori *lipopolysaccharides and human gastric epithelial cell surface glycoforms. J Biol Chem.

[B6] Monteiro MA, Appelmelk BJ, Rasko DA, Moran AP, Hynes SO, MacLean LL, Chan KH, Michael FS, Logan SM, O'Rourke J, Lee A, Taylor DE, Perry MB (2000). Lipopolysaccharide structures of *Helicobacter pylori *genomic strains 26695 and J99, mouse model *H. pylori *Sydney strain, *H. pylori *P466 carrying sialyl Lewis X, and *H. pylori *UA915 expressing Lewis B classification of *H. pylori *lipopolysaccharides into glycotype families. Eur J Biochem.

[B7] Rasko DA, Keelan M, Wilson TJ, Taylor DE (2001). Lewis antigen expression by *Helicobacter pylori*. J Infect Dis.

[B8] Sherburne R, Taylor DE (1995). *Helicobacter pylori *expresses a complex surface carbohydrate, Lewis X. Infect Immun.

[B9] Merkx-Jacques A, Obhi RK, Bethune G, Creuzenet C (2004). The *Helicobacter pylori flaA1 *and *wbpB *genes control lipopolysaccharide and flagellum synthesis and function. J Bacteriol.

[B10] Salaün L, Snyder LA, Saunders NJ (2003). Adaptation by phase variation in pathogenic bacteria. Adv Appl Microbiol.

[B11] Saunders NJ, Henderson B, Oyston PCF (2003). Evasion of antibody responses: Bacterial phase variation. Bacterial Evasion of Host Immune Responses.

[B12] Salaün L, Linz B, Suerbaum S, Saunders NJ (2004). The diversity within an expanded and redefined repertoire of phase-variable genes in *Helicobacter pylori*. Microbiology.

[B13] Salaün L, Ayraud S, Saunders NJ (2005). Phase variation mediated niche adaptation during prolonged experimental murine infection with *Helicobacter pylori*. Microbiology.

[B14] Falush D, Wirth T, Linz B, Pritchard JK, Stephens M, Kidd M, Blaser MJ, Graham DY, Vacher S, Perez-Perez GI (2003). Traces of human migrations in *Helicobacter pylori *populations. Science.

[B15] Wirth T, Wang X, Linz B, Novick RP, Lum JK, Blaser M, Morelli G, Falush D, Achtman M (2004). Distinguishing human ethnic groups by means of sequences from *Helicobacter pylori*: lessons from Ladakh. Proc Natl Acad Sci USA.

[B16] Suerbaum S, Smith JM, Bapumia K, Morelli G, Smith NH, Kunstmann E, Dyrek I, Achtman M (1998). Free recombination within *Helicobacter pylori*. Proc Natl Acad Sci USA.

[B17] Achtman M, Azuma T, Berg DE, Ito Y, Morelli G, Pan ZJ, Suerbaum S, Thompson SA, van der Ende A, van Doorn LJ (1999). Recombination and clonal groupings within *Helicobacter pylori *from different geographical regions. Mol Microbiol.

[B18] Falush D, Kraft C, Taylor NS, Correa P, Fox JG, Achtman M, Suerbaum S (2001). Recombination and mutation during long-term gastric colonization by *Helicobacter pylori*: estimates of clock rates, recombination size, and minimal age. Proc Natl Acad Sci USA.

[B19] Smith JM, Smith NH, O'Rourke M, Spratt BG (1993). How clonal are bacteria?. Proc Natl Acad Sci USA.

[B20] Tomb JF, White O, Kerlavage AR, Clayton RA, Sutton GG, Fleischmann RD, Ketchum KA, Klenk HP, Gill S, Dougherty BA, Nelson K, Quackenbush J, Zhou L, Kirkness EF, Peterson S, Loftus B, Richardson D, Dodson R, Khalak HG, Glodek A, McKenney K, Fitzegerald LM, Lee N, Adams MD, Hickey EK, Berg DE, Gocayne JD, Utterback TR, Peterson JD, Kelley JM, Cotton MD, Weidman JM, Fujii C, Bowman C, Whatthey L, Wallin E, Hayes WS, Borodovsky M, Karp PD, Smith HO, Fraser CM, Venter JC (1997). The complete genome sequence of the gastric pathogen *Helicobacter pylori*. Nature.

[B21] Alm RA, Ling LS, Moir DT, King BL, Brown ED, Doig PC, Smith DR, Noonan B, Guild BC, deJonge BL, Carmel G, Tummino PJ, Caruso A, Uria-Nickelsen M, Mills DM, Ives C, Gibson R, Merberg D, Mills SD, Jiang Q, Taylor DE, Vovis GF, Trust TJ (1999). Genomic-sequence comparison of two unrelated isolates of the human gastric pathogen *Helicobacter pylori*. Nature.

[B22] Lee A, O'Rourke J, De Ungria MC, Robertson B, Daskalopoulos G, Dixon MF (1997). A standardized mouse model of *Helicobacter pylori *infection: introducing the Sydney strain. Gastroenterology.

[B23] Bonfield JK, Smith K, Staden R (1995). A new DNA sequence assembly program. Nucleic Acids Res.

[B24] Thompson JD, Higgins DG, Gibson TJ (1994). CLUSTAL W: improving the sensitivity of progressive multiple sequence alignment through sequence weighting, position-specific gap penalties and weight matrix choice. Nucleic Acids Res.

[B25] Nei M, Gojobori T (1986). Simple methods for estimating the numbers of synonymous and nonsynonymous nucleotide substitutions. Mol Biol Evol.

[B26] Pride DT, Blaser MJ (2002). Concerted evolution between duplicated genetic elements in *Helicobacter pylori*. J Mol Biol.

[B27] Saitou N, Nei M (1987). The neighbor-joining method: a new method for reconstructing phylogenetic trees. Mol Biol Evol.

[B28] Kimura M (1980). A simple method for estimating evolutionary rates of base substitutions through comparative studies of nucleotide sequences. J Mol Evol.

[B29] Felsenstein J (1985). Confidence limits on phylogenies: an approach using the boostrap. Evolution.

[B30] Kumar S, Tamura K, Jakobsen IB, Nei M (2001). MEGA2: molecular evolutionary genetics analysis software. Bioinformatics.

