# Supplementary material for: Population-associated differences between the phase variable LPS biosynthetic genes of Helicobacter pylori
Source: BMC Microbiol. 2006 Sep 18;6:79. doi: 10.1186/1471-2180-6-79 (PMC1599737; doi:10.1186/1471-2180-6-79)
Supplement: Additional file 1 — Primers used for amplification and sequencing of the LPS biosynthesis genes under study. This file contains the sequences of all the primers used in the study [file 1471-2180-6-79-S1.doc]

**Additional data file**

Table S1: Primers used for amplification and sequencing of the LPS biosynthesis genes under study

For PCR, pairs of primers were used as following: (HPxxxx_WF1 - HPxxxx_WR1), (HPxxxx_WF2 - HPxxxx_WR1), (HPxxxx_WF1 - HPxxxx_WR2), and (HP0619tandemF - HP0619_WR1)

HPxxxx_WF1 and HPxxxx_WR1 were designed to regions located within the coding regions of the adjacent genes, and HPxxxx_WF2 and HPxxxx_WR2 were designed to regions located within the coding region of the genes under study

* for some genes, PCR amplification and sequencing needed primers specifically designed for a given strain
